# ABCC10 Plays a Significant Role in the Transport of Gefitinib and Contributes to Acquired Resistance to Gefitinib in NSCLC

**DOI:** 10.3389/fphar.2018.01312

**Published:** 2018-11-20

**Authors:** Hongbo Zhao, Yutang Huang, Jingjing Shi, Yi Dai, Lanxiang Wu, Honghao Zhou

**Affiliations:** ^1^Shanghai Key Laboratory of Female Reproductive Endocrine Related Diseases, Obstetrics and Gynecology Hospital, Fudan University, Shanghai, China; ^2^Institute of Life Sciences, Chongqing Medical University, Chongqing, China; ^3^Pharmacogenetics Research Institute, Institute of Clinical Pharmacology, Central South University, Changsha, China

**Keywords:** ABC transporter, ABCC10, gefitinib, acquired resistance, non-small cell lung cancer

## Abstract

Gefitinib, an epidermal growth factor receptor (EGFR) tyrosine kinase inhibitor (EGFR-TKI), is used clinically as first-line therapy in patients with advanced non-small cell lung cancer (NSCLC) with EGFR activating mutations, but the inevitable development of acquired resistance limits its efficacy. In up to 30–40% of NSCLC cases, the mechanism underlying acquired resistance remains unknown. ATP-binding cassette (ABC) transporters are a family of membrane proteins that can significantly influence the bioavailability of numerous drugs, and have confirmed to play an essential role in multidrug resistance (MDR) in cancer chemotherapy. However, their role in acquired resistance to gefitnib in NSCLC has not been well studied. Here, through RNA sequencing (RNA-Seq) technology we assessed the differentially expressed ABC transporters in gefitinib-sensitive (PC9 and H292) and gefitinib-resistant (PC9/GR and H292/GR) NSCLC cells, with ABCC10 identified as a transporter of interest. Both ABCC10 mRNA and protein were significantly increased in acquired gefitinib-resistant NSCLC cells, independent of EGFR mutation status. *In vitro* transport assay showed that ABCC10 could actively efflux gefitinib, with an efflux ratio (ER) of 7.8. Further results from *in vitro* cell line models and *in vivo* xenograft models showed that overexpression of ABCC10 led to a reduction in gefitinib sensitivity through decreasing the intracellular gefitinib accumulation. Our data suggest that ABCC10 has an important role in acquired resistance to gefitinib in NSCLC, which can serve as a novel predictive marker and a potential therapeutic target in gefitinib treatment.

## Introduction

Lung cancer is by far the leading cause of cancer-related deaths in both men and women (Ridge et al., 2013). Non-small cell lung cancer (NSCLC) represents approximately 85% of all lung cancer case, with 5-year survival rates of only 16% (Tas et al., 2013). In 40–80% of NSCLCs, the epidermal growth factor receptor (EGFR) has been found to be overexpressed, which is believed to contribute to the NSCLC cell proliferation, differentiation, and survival (Ennis et al., 1991; Salomon et al., 1995; Jutten and Rouschop, 2014). In 2015, gefitinib, one of the first-generation EGFR tyrosine kinase inhibitors (EGFR-TKIs), was approved by the United States Food and Drug Administration (USFDA) as a first-line treatment for metastatic NSCLC patients with activating EGFR mutations, resulting a longer progression-free survival (PFS) compared to standard cytotoxic chemotherapy (Gridelli et al., 2011; Douillard et al., 2014). Besides, previous studies also support that a considerable proportion of NSCLC patients harboring wild-type EGFR still achieve clinical benefit from gefitinib treatment (Zhou and Zhou, 2015; Shepherd, 2016).

Although gefitinib has a favorable clinical response, almost all patients will eventually develop acquired resistance after 10–14 months, which finally leads to treatment failure (Gandara et al., 2014). To date, many mechanisms of acquired resistance to gefitinib have been defined in around 60–70% of cases, which can be classified into three categories: secondary EGFR mutations, phenotypic transformation, and activation of alternative pathways (Morgillo et al., 2016). However, the mechanisms in about 30–40% of cases still remain unknown.

ATP-binding cassette (ABC) transporters are a family of membrane proteins that pump out of the cells a variety of endogenous and exogenous substrates in an ATP-dependent manner (Schinkel and Jonker, 2003). Nearly 40 years of research indicates that ABC-mediated multidrug efflux is one of the most important mechanisms of multidrug resistance (MDR), a major obstacle in the clinical treatment of various cancers. Due to the strong correlation between ABC transporters and MDR, a number of ABC inhibitors have been developed and tested in clinical trials. Unfortunately, the majority of trials did not confirm clinical benefit. There are multiple reasons for these negative results, but one of the main reason is the insufficient knowledge of the interactions between ABC transporters and chemotherapy drugs (Jaramillo et al., 2018; Mohammad et al., 2018; Robey et al., 2018). Owing to considerable overlap among the substrate profiles of the various ABC transporters, multiple transporters may be involved in the efflux of a specific chemotherapy agent. Therefore, it is critical that all known ABC transporters be studied within a single study, rather than focusing on individuals (Tamaki et al., 2011). In the case of gefitinib, only its interactions with ATP binding cassette transporter G2 (ABCG2), ATP binding cassette transporter B1 (ABCB1), and ATP binding cassette transporter C1 (ABCC1) have been examined previously. ABCG2 has been shown to be a high affinity gefitinib transporter, at least at submicromolar concentrations, and likely play an important role in acquired resistance to gefitinib (Leggas et al., 2006; Usuda et al., 2007; Azzariti et al., 2010; Chen et al., 2011; Hegedüs et al., 2012; Zhu et al., 2015). While ABCB1 and ABCC1 exhibit much lower affinities toward gefitinib (Ozvegy-Laczka et al., 2004; Beretta et al., 2017). However, no investigations into the interaction between other ABC transporters and gefitinib have been conducted so far.

In the present study, we mapped the expression of 48 human ABC transporters in the gefitinib-sensitive and -resistant NSCLC cell lines using RNA sequencing technology, and found that ABCC10 (ATP binding cassette subfamily C member 10), also known as MDR protein 7 (MRP7), was significantly upregulated in cells with acquired resistance to gefitinib. Results from *in vitro* cell culture models and *in vivo* xenograft models showed that ABCC10 could actively pump gefitinib out of cells, and its overexpression led to a reduction in gefitinib sensitivity through decreasing the intracellular gefitinib accumulation.

ABCC10 is an important member of ABC transporter superfamily. Accumulating research has revealed that ABCC10 actively transports a broad range of cytotoxic chemotherpy agents, such as taxanes, vinca alkaloids, antifolates, cisplatin, daunorubicine, etoposide, irinotecan, epothilone B, as well as nucleoside analogs, leading to the occurrence of MDR (Wu et al., 2016; Dabrowska and Sirotnak, 2017). Additionally, ABCC10 may interact with some EGFR-TKIs. A recent study has shown that lapatinib and erlotinib reverse ABCC10-mediated MDR through inhibition of the drug efflux function (Kuang et al., 2010). Here, our data suggest that ABCC10 has an important role in acquired resistance to gefitinib in NSCLC, which can serve as a novel predictive marker and a potential therapeutic target in gefitinib treatment.

## Materials and methods

### Cell lines and cultures

The EGFR-mutant PC9 (exon 19 deletion E746-A750) and EGFR wild-type H292 NSCLC cell lines, as well as Lewis lung carcinoma-porcine kidney epithelial cell line (LLC-PK1) were purchased from the Cellular Institute of Chinese Academy of Science. NSCLC cell lines were cultured with RPMI 1640 supplemented with 10% fetal bovine serum (FBS), and LLC-PK1 cells were maintained in complete Dulbecco's modified Eagle's medium (DMEM) supplemented with 10% FBS. All the cell lines were cultured in a 5% CO_2_ incubator at 37°C.

To establish acquired gefitinib-resistant cell lines PC9/GR and H292/GR, PC9 and H292 cells were continuously exposed to increasing dosages of gefitinib for ~12 months. Established resistant cell lines were maintained by culture in a medium containing 2 μmol/L gefitinib. To eliminate the effects of gefitinib, the resistant cells were cultured in a drug-free medium for at least 2 weeks before all experiments.

### Establishment of stable cell lines

The human *ABCC10* or *ABCG2* gene was inserted into the EcoRI and XbaI sites of pcDNA3.1(+) (Invitrogen, Carlsbad, CA) to make expression vectors, pcDNA3.1(+)/*ABCC10* or pcDNA3.1(+)/*ABCG2*.

To establish ABCC10-overexpressing NSCLC cells, gefitinib-sensitive PC9 and H292 cells were transfected with pcDNA3.1(+)/*ABCC10* or empty vector using Lipofectamine^TM^ 2000 (Invitrogen, Carlsbad, CA, USA). To establish NSCLC cells with ABCC10 knockdown, *ABCC10* shRNA plasmid (shABCC10, Santa Cruz Biotechnology, sc-62641-SH) or control plasmid (shMock, Santa Cruz Biotechnology, sc-108060) was introduced into gefitinib-resistant NSCLC cells PC9/GR and H292/GR. Single colonies were identified in culture medium containing G418 (2 mg/mL) and subcultured for further analysis.

To establish the stably transfected LLC-PK1 cells expressing ABCC10 or ABCG2, pcDNA3.1(+)/*ABCC10*, or pcDNA3.1(+)/*ABCG2* was transfected into LLC-PK1 cells, and stable transfected clones were selected as described above. Expression of ABCC10/ABCG2 was confirmed by quantitative real-time PCR and western blot analysis as described below.

### Cell viability assay

Cell viability was measured using the CellTiter96 Aqueous One Solution Cell Proliferation Assay (MTS) (Promega, Madison, WI, United States). In brief, cells were plated in 96-well plates at the density of 2 × 10^4^ cells per well. After 24 h incubation, cells were treated with various concentrations of gefitinib (0.1–10 μmol/L) for 72 h. Then, the 20 μL of MTS reagent was added to each well and the plates were incubated for an additional 2 h. The absorbance was read at 490 nm using a microplate reader (SynergyTMH4, BioTek, United States). Cell viability was calculated as a percentage relative to vehicle-treated control. the IC_50_ value was calculated based on the non-linear regression fit method by Graphpad Prism 4.0 software (San Diego, CA).

### Cell apoptosis assay

For apoptosis assay by flow cytometry, cells were seeded in 6-well plates at a concentration of 2 × 10^5^ cells per well, and treated with 1 μmol/L gefitinib for 72 h. Cells were then digested with trpsin and washed with PBS three times, incubated with 5 μL of FITC-conjugated Annexin-V and 5 μL of propidium iodide (PI) (Thermo Fisher Scientific, MA, United States) for 15 min in a dark place at room temperature. The stained cells were detected using the BD Accuri C6 flow cytometer (BD Biosciences, CA, United States). At least 10,000 cells were analyzed for each group.

### RNA isolation and RNA sequencing (RNA-seq) analysis

Total RNA was isolated from 1 × 10^7^ cells using the mirVana™ miRNA ISOlation Kit (Ambion, Austin, TX, United States) following the manufacturer's instruction. 1 μg of total RNA was used to prepare standard RNA-seq libraries (TruSeq Stranded Total RNA kit with Ribo-Zero Gold, Illumina). RNA integrity was validated for size using the Agilent 2100 Bioanalyzer (Agilent Technologies, CA, United States) and sequenced by a 2 × 125 bp paired-end sequencing module on an Illumina HiSeq 2500 (Oebiotech, Shanghai, China). The criteria for differential gene expression included a fold change ≥2 between compared groups and statistical significance at *P* < 0.05.

### Reverse transcription and quantitative real-time PCR

Total RNA was isolated from the cells using TRIzol reagent (Invitrogen, Carlsbad, CA, United States), and the first strand cDNA was synthesized using the Revert Aid TM H Minus First Strand cDNA Synthesis Kit (TaKaRa Bio, Shiga, Japan) according to the manufacturer's instruction. Quantitative real-time PCR (qRT-PCR) was performed using SYBR Premix Ex Taq II (Takara Bio, Shiga, Japan) according to the manufacturer's instructions. The sequences of the *ABCC10* primers were 5′-CGGGTTAAGCTTGTGACAGAGC-3′ (forward) and 5′-AACACCTTGGTGGCAGTGAGCT-3′ (reverse). PCR programs were carried out as follows: 95°C for 30 s, followed by 40 cycles of 95°C for 5 sec, 60°C for 30 s. β*-actin* served as an internal control. Relative quantification of *ABCC10* was analyzed using the 2^−ΔΔ*Ct*^ method as a ratio relative to the β*-actin* expression level in each sample (Livak and Schmittgen, 2001).

### Protein isolation and western blot analysis

Crude membrane fraction from cells was isolated as described previously (Revalde et al., 2015). Total protein was extracted using RIPA buffer (Beyotime, Shanghai, China), with PMSF (Sigma, Missouri, United States). The Pierce BCA Protein Assay Kit (Thermo Fisher Scientific, Waltham, United States) was used to determine the protein concentration. The protein extracts were separated by 10% sodium dodecyl sulfate-polyacdene gel electrophoresis and transferred onto polyvinylidene difluoride membranes (Bio-Rad, Hercules, CA, United States). The membranes were incubated with anti-ABCC10, anti-ABCG2, anti-β-actin, and anti-Na^+^/K^+^-ATPase primary antibodies (Abcam, Cambridge, United Kingdom) at 4°C overnight, probed with horseradish peroxidase-conjugated secondary antibodies (Abcam, Cambridge, United kingdom), and signals were detected by ECL^TM^ Prime (GE Healthcare, Buckinghamshire, United Kingdom) and a LAS-3000 imager (Fujifilm, Tokyo, Japan). β-actin and Na^+^/K^+^-ATPase expression levels were used to normalize the total and membrane expression levels of ABCC10.

### Measurement of intracellular gefitinib accumulation assay

Concentrations of gefitinib accumulated in cells were determined using a validated Liquid chromatography tandem mass spectrometry (LC-MS/MS) method. The cells were cultured in the 6-well plates at a density of 1 × 10^6^ per well and were grown to 85% confluence. Then, the cells were treated with varying concentration of gefitinib with or without 2.5 μmol/L cepharanthine at 37°C for 4 h, harvested and washed in cold PBS. Gefitinib was extracted from cells with 200 μL of a methanol/water mixture (50/50, v/v), cell extracts were centrifuged (4°C, 12,000 rpm/min, 10 min) and collected. Samples were prepared by adding 20 μL of internal standard solution (100 ng/mL, erlotinib) to 180 μL of the cell lysates. Cell proteins were quantified using the BCA Protein Assay kit (Thermo Scientific, Rockford, IL, United States). LC-MS/MS analysis was performed on an Agilent 1290 series liquid chromatography system (Agilent Technologies, Palo Alto, CA, United States) and an Agilent 6470 triple-quadruple mass spectrometer (Agilent Technologies, Santa Clara, CA, United States). An Agilent ZORBAX Eclipse Plus C18 column (1.8 μm, 3.0 mm × 50 mm) and a mobile phase [water (containing 0.1% formic acid) and methanol (30:70, v:v)] at a flow rate of 3 mL/min were applied. The ion transitions monitored were as follows: m/z 447.1 ([M+H]^+^) to 128.4 ([M+H]^+^) for gefitinib, and m/z 394.2 ([M+H]^+^) to 336.1 ([M+H]^+^) for erlotinib. The running time of each sample was 3 min. The lower limit of quantification for gefitinib was 1 ng/mL. The calibration curves were linear over the rage 1–500 ng/mL with mean correlation coefficients of 0.9997. The intra- and inter-day coefficients of variation were < 10%.

### LLC-PK1 monolayer transport assay

Polarized LLC-PK1 cells were used in transport assay. Wildtype LLC-PK1 (LLC–WT) cells, human *ABCC10* or *ABCG2* gene transfected LLC-PK1 (LLC-ABCC10 or LLC-ABCG2) cells were seeded on the permeable polycarbonate Transwell® cell culture inserts (24 mm diameter, 0.4 μm pore size; Costar, MA, United States) at a density of 2 × 10^6^ per well for 72 h. Before the start of transport assay, cells were washed with PBS and preincubated with Opti-MEM (Invitrogen, Carlsbad, CA, United States) for 2 h, then the experiment was stated by replacing the medium on either the apical or the basal side of the cell layer with fresh DMEM containing 10% fetal calf serum and 10 nmol/L [^3^H]-estrone-3-sulfate, 100 nmol/L [^3^H]-paclitaxel, or 100 nmol/L [^3^H]-gefitinib with or without 2.5 μmol/L cepharanthine or 10 μmol/L Ko143. The cells were incubated at 37°C under 5% CO_2_. After various time of incubation, 100 μL of medium was taken from each compartment, and the radioactivity in each aliquot was measured in a liquid scintillation counter (LS6500; Beckman Coulter, Inc., Fullerton, CA, United States). Immediately after each of the experiments, fluorescein isothiocyanate (FITC)-conjugated dextran (MW = 40 kDa) was used to examine the integrity of the cell monolayer. Leakage of dextran had to remain < 1% of the total added radioactivity per hour. The apparent permeability (*P*_app_) was calculated by the following equation

(1) $$\begin{array}{r} P_{\text{app}}=\frac{dQ/dt}{A\times C_{0}} \end{array}$$

where *Q* is the amount of radioactivity transported across the monolayer, *t* is time, *dQ/dt* is the rate of transport, *A* is the effective surface area of the cell monolayer, and *C*_0_ is the initial drug concentration. The efflux ratio (ER) was used as a meausre of ABCC10/ABCG2-mediated efflux, and calculated according to Equation 2:

(2) $$\begin{array}{r} \text{ER}=\frac{P_{\text{app}}\text{B}\to \text{A}}{P_{\text{app}}\text{A}\to \text{B}} \end{array}$$

where *P*_app__B → *A*_ is the *P*_app_ value measured in the basal to apical direction, and *P*_app__A → *B*_ is the *P*_app_ value measured in the apical to basal direction (Shaik et al., 2007).

### NSCLC xenograft mice models

The protocols of animal experiments were approved by the Animal Ethics and Experimental Committee of the Chongqing Medical University (Chongqing, China), and performed according to the National Institutes of Health Guide for the Care and Use of Laboratory Animals. Briefly, female Balb/c-nude mice (4–6 weeks, 16–20 g) were obtained from the Laboratory Animal Center of Chongqing Medical University (Chongqing, China), and randomized into four groups (*n* = 6). Mice subjected to subcutaneously injection with ABCC10-overexpressing PC9 cells (PC9-ABCC10), empty-vector transfected PC9 cells (PC9-EV), ABCC10-knockdown PC9/GR cells (PC9/GR-shABCC10), or shMock-transfected PC9/GR cells (PC9/GR-shMock) (5 × 10^6^ cells/mouse) in each right flank. When all tumors reached a mean volume of 50 mm^3^, the mice were treated with gefitinib (30 mg/kg/day) for 3 weeks by oral gavage. Tumor volume (TV) was calculated as formula: TV (mm^3^) = (L × W^2^)/2 (L, lone diameter; W, wide diameter). Body weights were recorded every 3 days. At the end of experiments, mice were sacrificed and the tumors were removed.

### Immunohistochemical staining

PC9 xenografts were collected, fixed in 4% paraformaldehyde, embedded in paraffin. Slides from each group were deparaffinized in xylene, and incubated with anti-Ki-67 antibodies (Abcam, Cambridge, United kingdom). The primary antibody was detected with a biotinylated goat anti-rabbit IgG. Three slides per groups were read and scored for the number of Ki-67-positive cells using the Visiomorph Integrator image analysis system. The proliferating cells were estimated by the percentage of Ki-67 positive-stained cells in 10 randomly chosen high-powered fields for each section (× 400).

### Statistical analysis

Statistical analysis was performed using the SPSS 20.0 software (IBM SPSS, Armonk, NY, United States). Heat map was performed by Clustering (version 3.17.1). All values were expressed as mean ± SEM. One-way ANOVA and subsequent *post hoc* Tukey's test were performed to analyze the differences between sets of data. Values of *P* < 0.05 were considered statistically significant.

## Results

### Gene expression profile of ABC transporters in gefitinib-sensitive and -resistant NSCLC cell lines

**Figure 1:**
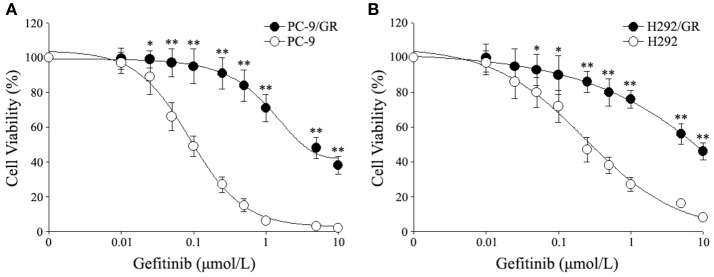
Cytotoxic effects of gefitinib in acquired gefitinib-resistant NSCLC cell lines and their parental cells. PC9, PC9/GR **(A)** and H292, H292/GR **(B)** cells were cultured in 96-well plates and exposed to the indicated concentrations of gefitinib for 72 h. The cell viability was analyzed using MTS assay as described in Meterials and Methods. The number of viable cells is expressed as a percentage of the value for untreated cells. *P < 0.05, **P < 0.01 compared to the parent-sensitive cell line.

In order to explore a new mechanism of acquired resistance to gefitinib in NSCLC, the acquired gefitinib-resistant cell lines that were derived from the parental sensitive PC9 and H292 cells were established through continuous exposure of this drug, which were designated as PC9/GR and H292/GR. MTS proliferation assay was used to confirm the acquired resistance to gefitinib. The IC_50_ values for gefitinib was 0.15 ± 0.03 μmol/L in PC9 cells, while PC9/GR cells showed about 32-fold resistance to gefitinib, with the IC_50_ value of 4.65 ± 0.84 μmol/L. Similarly, the IC_50_ was 1.01 ± 0.22 μmol/L in H292 cells, and the H292/GR showed about 11-fold higher resistance to gefitinib, with the IC50 value of 10.90 ± 1.8 μmol/L (Figure 1).

**Figure 2:**
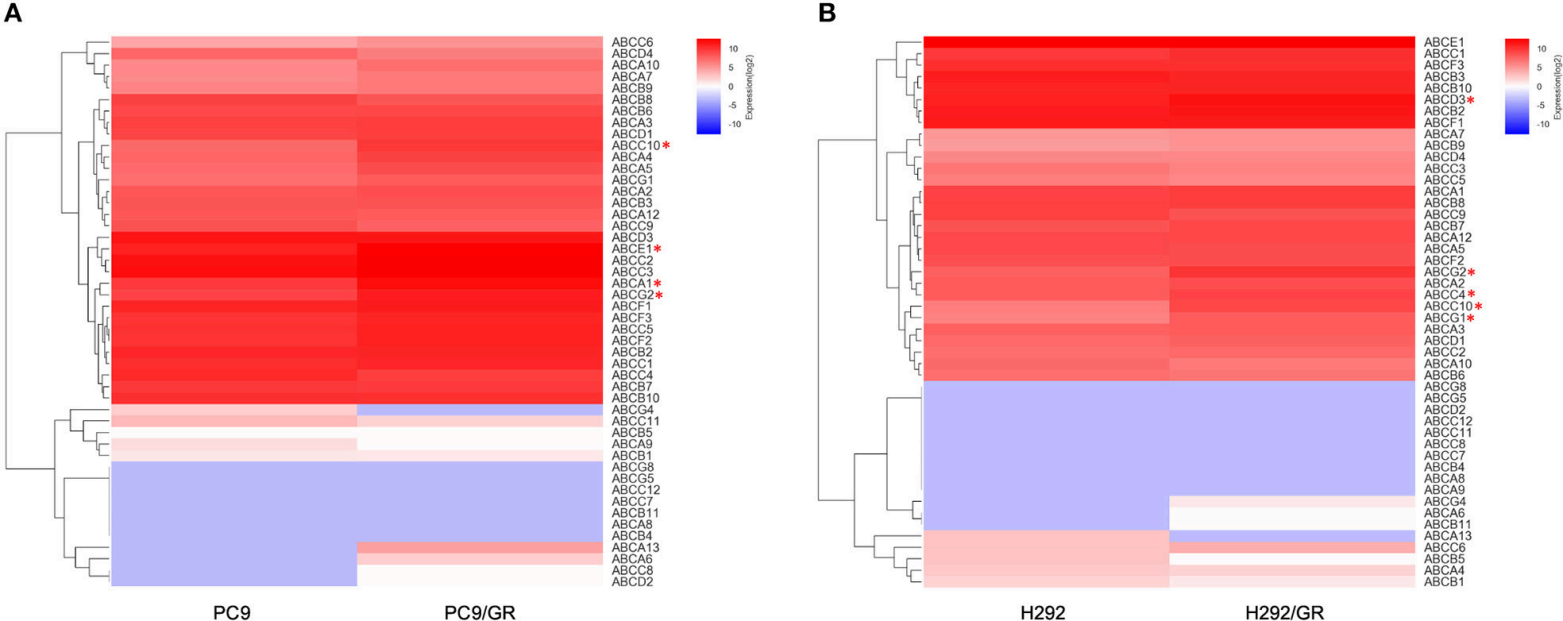
Differentially expressed genes encoding ABC transporters between gefitinib-sensitive and -resistant cell lines. **(A)** Analysis of differentially expressed genes between PC9 and PC9/GR cell lines. **(B)** Analysis of differentially expressed genes between H292 and H292/GR cell lines. Expression intensities are displayed from blue (low expression) to red (high expression). *P < 0.05 compared to the parent-sensitive cell line.

The gene expression profile of 48 human ABC transporters in gefitinib-resistant PC9/GR and H292/GR cells were examined using RNA-seq and compared with that of the parental cell lines. The changes in transcription level of 48 genes encoding ABC transporters in the gefitinib-sensitive and -resistant NSCLC cell lines were shown in Supplementary Tables 1, 2. The reliability and the reproducibility between assays were assessed by repeating the experiments three times for each cell line (*n* = 3). The transcripts with a greater than 2-fold change in expression and an adjusted two-sided *P* < 0.05 were considered to have significantly differential expression between two groups. Figure 2 shows the data for one representative experiment. Compared to their parental cells, four genes encoding *ABCA1, ABCC10, ABCG2*, and *ABCE1* were significantly upregulated in PC9/GR cells, and five genes encoding *ABCC4, ABCC10, ABCD3, ABCG2*, and *ABCG1* were significantly upregulated in H292/GR cells. Therefore, the present results showed that except for *ABCG2*, a transporter that is known to be responsible for the acquired resistance to gefitinib in NSCLC (Leggas et al., 2006; Usuda et al., 2007; Azzariti et al., 2010; Chen et al., 2011; Hegedüs et al., 2012; Zhu et al., 2015), *ABCC10* was the only significant differential expressed transporter in both PC9/GR and H292/GR cells.

### ABCC10 is overexpressed in gefitinib-resistant NSCLC cell lines

**Figure 3:**
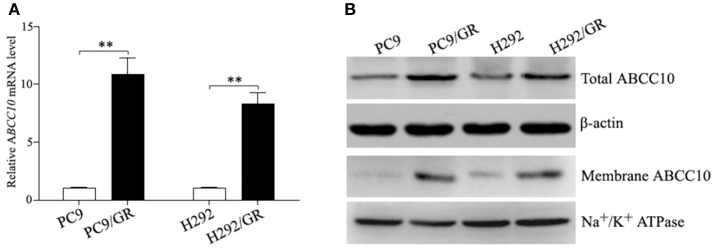
ABCC10 expression in gefitinib-sensitive and -resistant NSCLC cell lines. **(A)** qRT-PCR analysis of *ABCC10* mRNA levels in gefitinib-sensitive and -resistant NSCLC cell lines. **(B)** Western blot analysis of ABCC10 protein levels in gefitinib-sensitive and -resistant NSCLC cell lines. **P < 0.01 compared to the parent-sensitive cell line.

To verify the sequencing result, we quantitated the expression of *ABCC10* mRNA in gefitinib-sensitive and -resistant NSCLC cell lines using qRT-PCR. Our results showed that the expression patterns observed in all cell lines were consistent with the RNA-seq results. Compared to their parent-sensitive cell lines, *ABCC10* mRNA levels were increased about 10.9-fold in PC9/GR cells, and 8.3-fold in H292/GR cells (Figure 3A). Then, we examined the levels of ABCC10 protein in the lysates prepared from total and membrane proteins isolated from these cell lines. Increased total and plasma membrane ABCC10 levels were observed in cells with acquired resistance to gefitinib. Moreover, compared to the whole cell lysates, the upregulation of ABCC10 level was more obvious in the plasma membrane of two resistant cells (Figure 3B).

### Influence of ABCC10 on gefitinib sensitivity in NSCLC cells

**Figure 4:**
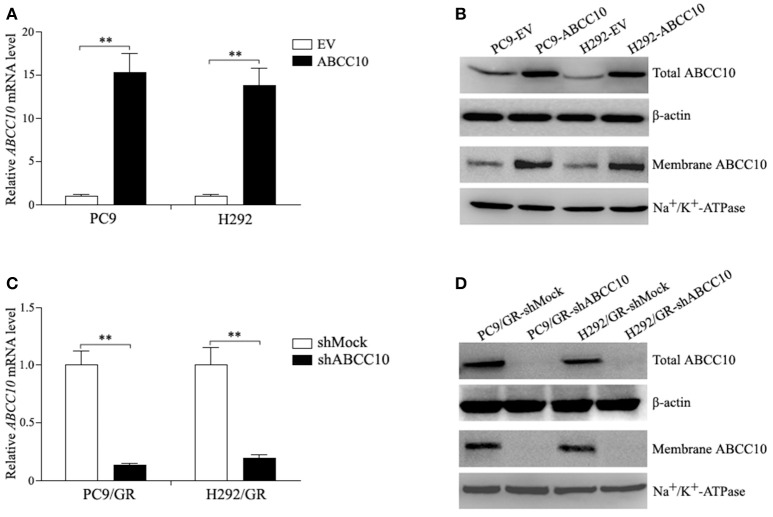
ABCC10 expression in gefitinib-sensitive and -resistant NSCLC cells following ABCC10 overexpression and knockdown. **(A,B)** ABCC10 mRNA and protein levels in gefitinib-sensitive PC9 and H292 cells following ABCC10 overexpression. **(C,D)** ABCC10 mRNA and protein levels in gefitinib-resistant PC9/GR and H292/GR cells following ABCC10 knockdown. **P < 0.01 compared to the empty-vector-transfected cells (EV) or shMock-transfected cells (shMock).

**Figure 5:**
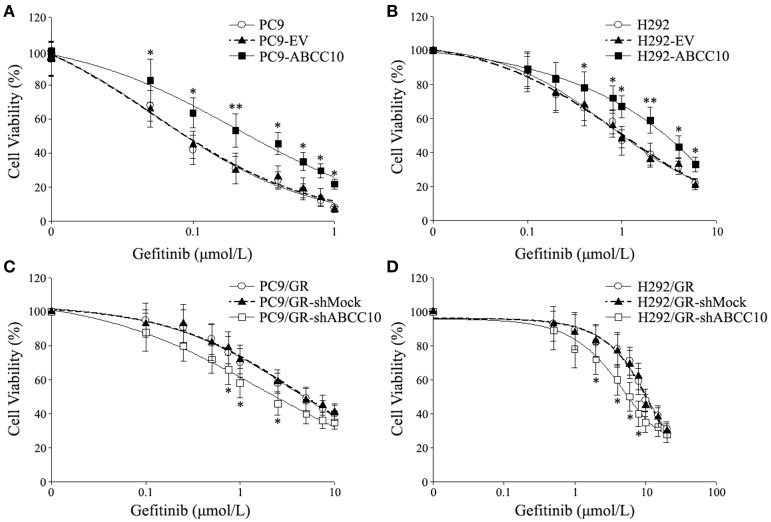
Effect of ABCC10 on cell viability of gefitinib-sensitive and -resistant NSCLC cells after gefitinib exposure. **(A,B)** Influence of ABCC10 overexpression on cell viability of gefitinib-sensitive PC9 and H292 cells treated with various concentration of gefitinib for 72 h. **(C,D)** Influence of ABCC10 knockdown on cell viability of gefitinib-resistant PC9/GR and H292/GR cells treated with various concentration of gefitinib for 72 h. *P < 0.05, **P < 0.01 compared to the empty-vector-transfected cells (EV) or shMock-transfected cells (shMock).

**Figure 6:**
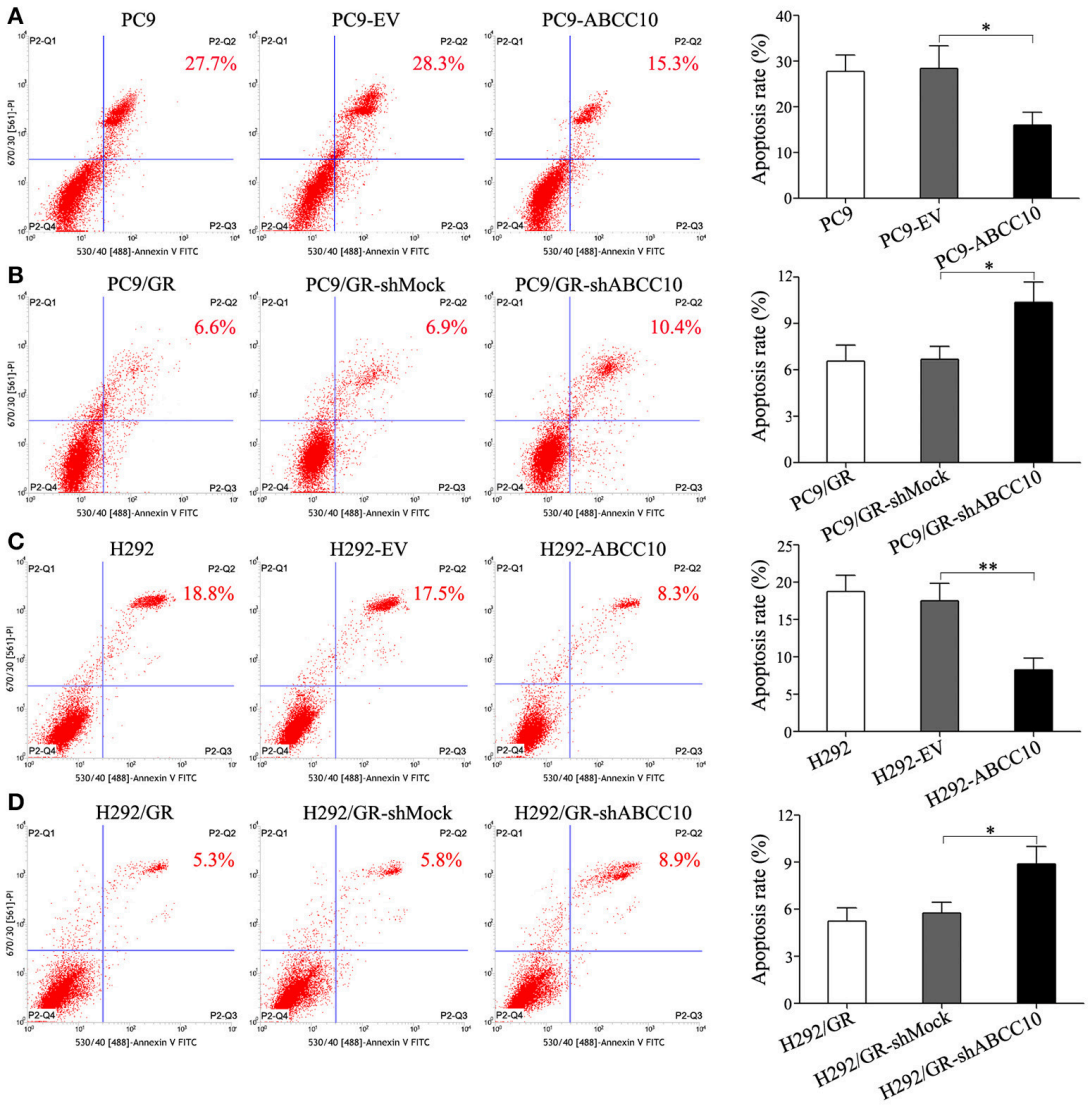
Effect of ABCC10 on cell apoptosis of gefitinib-sensitive and -resistant NSCLC cells after gefitinib exposure. **(A,C)** Influence of ABCC10 overexpression on cell apoptosis of gefitinib-sensitive PC9 and H292 cells treated with 1 μmol/L gefitinib for 72 h. **(B,D)** Influence of ABCC10 knockdown on cell apoptosis of gefitinib-resistant PC9/GR and H292/GR cells treated with 1 μmol/L gefitinib for 72 h. *P < 0.05, **P < 0.01 compared to the empty-vector-transfected cells (EV) or shMock-transfected cells (shMock).

To study the influence of ABCC10 on gefitinib sensitivity in NSCLC cells, we modulated the expression level of ABCC10 either by overexpression in gefitinib-sensitive NSCLC cells, or knockdown in gefitinib-resistant NSCLC cells (Figure 4), then observed the cytotoxic effect of gefitinib. Our results showed that compared with empty-vector-transfected cells, ABCC10 overexpression significantly increased the cell survival rate after 72 h of gefitinib exposure, the IC_50_ values were increased by 2.5-fold in PC9 cells, and 2.4-fold in H292 cells (Figures 5A,B). ABCC10 knockdown in gefitinib-resistant NSCLC cells caused about 38.5 and 34.1% reduction in IC_50_ values in PC9/GR and H292/GR cells, respectively (Figures 5C,D). In addition, after ABCC10 overexpression, gefitinib-induced apoptosis decreased from 27.7 to 15.3% in PC9 cells, and from 18.8 to 8.3% in H292 cells. Whereas ABCC10 knockdown led to increase rate of apoptosis from 6.6 to 10.4% in PC9/GR cells, and from 5.3 to 8.9% in H292/GR cells (Figure 6).

### Influence of ABCC10 on intracellular concentration of gefitinib in NSCLC cells

**Figure 7:**
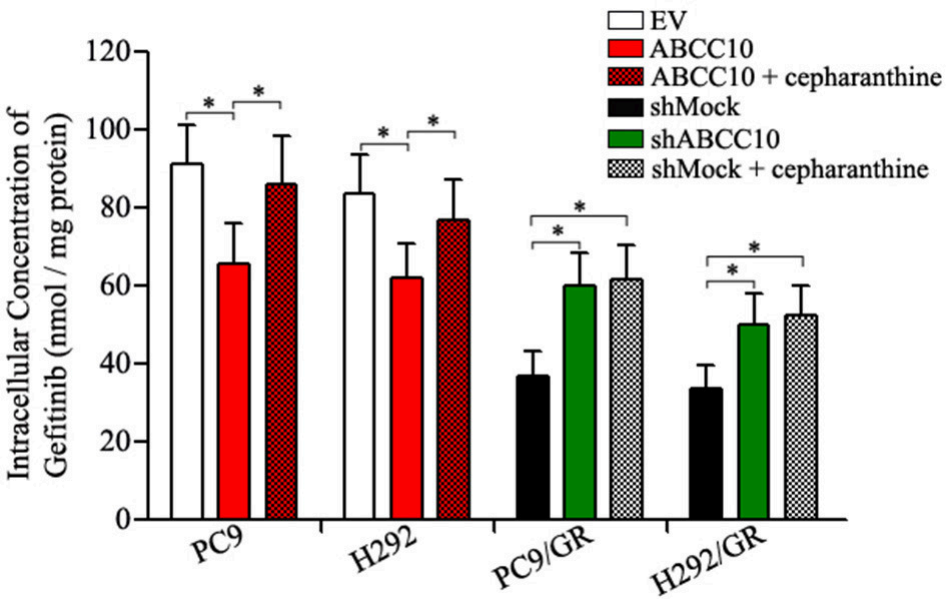
Effect of ABCC10 on intracellular gefitinib concentration in gefitinib-sensitive and –resistant NSCLC cells. cells were incubated with 1 μmol/L gefitinib with or without 2.5 μmol/L cepharanthine at 37°C for 4 h, then the intracellular gefitinib concentration was determined by LC-MS/MS assay and expressed as nmol/mg of protein. *P < 0.05 compared to the empty-vector-transfected cells (EV) or shMock-transfected cells (shMock).

The results above indicate that ABCC10 might play an important role in acquired resistance to gefitinib. Mechanistically, we detected the intracellular accumulation of gefitinib in NSCLC cells with different levels of ABCC10 expression. As shown in Figure 7, after ABCC10 overexpression, the intracellular gefitinib concentrations in gefitinib-sensitive PC9 and H292 cells were decreased by 25.3 and 23.7%, respectively. While after ABCC10 knockdown, the gefitinib-resistant cells showed higher intracellular concentration of gefitinib, 1.5-fold and 1.4-fold increases in PC9/GR and H292/GR cells, respectively. As expected, cepharanthine, an ABCC10 inhibitor (Zhou et al., 2009), could almost completely reverse the negative influence of ABCC10 on intracellular gefitinib accumulation (Figure 7).

### ABCC10-mediated gefitinib transport *in vitro*

**Figure 8:**
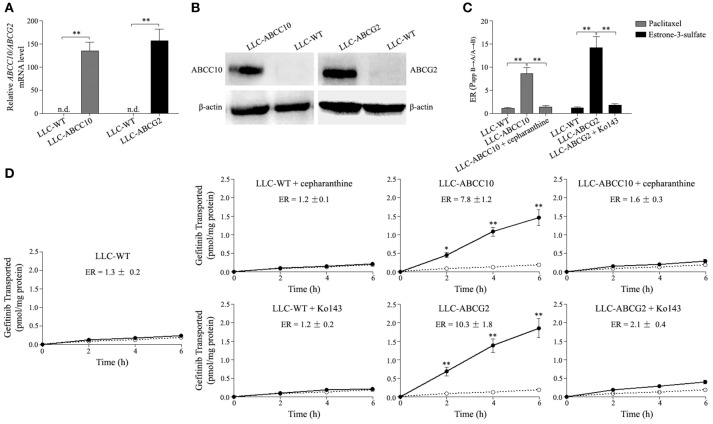
ABCC10 and ABCG2-mediated transport of gefitinib *in vitro*. **(A,B)** Characterization of the LLC-PK1 cell line overexpression human ABCC10 or ABCG2. mRNA and protein levels of human ABCC10 or ABCG2 were detected in wildtype LLC-PK1 (LLC-WT) cells, as well as its subclones transduced with human ABCC10 (LLC-ABCC10) or ABCG2 (LLC-ABCG2). **P < 0.01 compared with LLC-WT cells. **(C)**, Transepithelial transport of 100 nmol/L [^3^H]-paclitaxel and 10 nmol/L [^3^H]-estrone-3-sulfate in LLC-WT, LLC-ABCC10, and LLC-ABCG2 cells. At *t* = 0 h, [^3^H]-paclitaxel or [^3^H]-estrone-3-sulfate was applied in one compartment (apical or basal). At *t* = 4 h, the radioactivities were measured and the efflux ratios (ERs) were caluculated. **P < 0.01 compared with LLC-WT cells. **(D)** Transepithelial transport of 100 nmol/L [^3^H]-gefitinib was assessed in LLC-PK1 cells. At *t* = 0 h, [^3^H]-gefitinib was applied in one compartment (apical or basal), and the radioactivities at *t* = 2, 4, and 6 h were measured and plotted over time. Closed circles, translocation from the basal to the apical compartment; open circles, translocation from the apical to the basal compartment. *P < 0.05, **P < 0.01 compared to the translocation from the apical to the basal compartment.

As mentioned above, ABCC10 overexpression resulted in a decrease in the intracellular accumulation of gefitinib. Therefore, we evaluated whether gefitinib was a substrate of ABCC10 *in vitro*. As gefitinib is also a substrate of ABCG2, at least in the submicromolar concentration (Ozvegy-Laczka et al., 2004; Leggas et al., 2006; Azzariti et al., 2010; Hegedüs et al., 2012; Beretta et al., 2017), the ABCG2-mediated efflux of gefitinib was simultaneously observed in this study. Monolayers of wildtype LLC-PK1 (LLC-WT) cells, as well as its subclones transduced with human *ABCC10* (LLC-ABCC10) or *ABCG2* (LLC-ABCG2) were used in this study (Figures 8A,B). The polarized LLC-ABCC10 and LLC-ABCG2 cells predominantly exported [^3^H]-paclitaxel and [^3^H]-estrone-3-sulfate, two typical substrates for ABCC10 and ABCG2 (Hopper-Borge et al., 2004; Nakayama et al., 2011), from the basal to apical side, which indicates that both ABCC10 and ABCG2 localize mainly to the apical membrane (Figure 8C). As for gefitinib, there was no significant difference in its permeability in either direction in LLC-WT cells. However, the basal-to-apical permeability of gefitinib in the LLC-ABCC10 and LLC-ABCG2 cells were significantly greater than the apical-to-basal permeability, yielding ER values of 7.8 and 10.3, respectively. The directional transport was abolished when the cells were treated with 2.5 μmol/L cepharanthine (ABCC10 inhibitor) or 10 μmol/L Ko143 (ABCG2 inhibitor), indicating that, in addition to ABCG2, gefitinib is a good substrate of human ABCC10 (Figure 8D).

**Figure 9:**
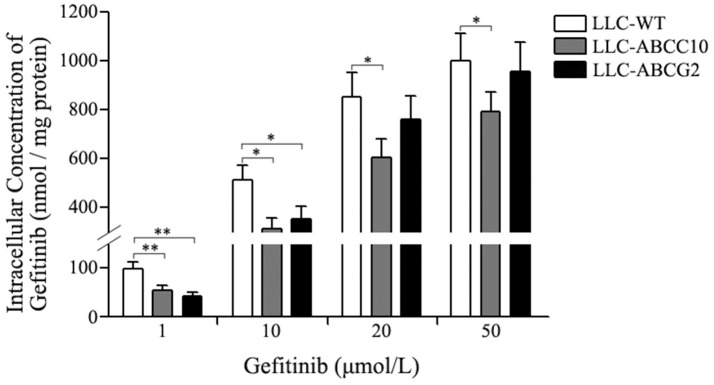
Intracellular gefitinib concentration in LLC-PK1 cells either wildtype (LLC-WT) or transduced with human *ABCC10* (LLC-ABCC10) or *ABCG2* (LLC-ABCG2) cDNA. cells were incubated with 1, 10, 20, and 50 μmol/L gefitinib at 37°C for 4 h, then the intracellular gefitinib concentration was determined by LC-MS/MS assay and expressed as nmol/mg of protein. *P < 0.05, **P < 0.01 compared to the LLC-WT cells.

We further observed the ABCC10 and ABCG2-mediated gefitinib efflux activity in the presence of various substrate concentration using LLC-WT, LLC-ABCC10, and LLC-ABCG2 cells. After incubation with varying concentrations of gefitinib for 4 h, the intracellular gefitinib accumulation was measured by LC-MS/MS assay. The results showed that LLC-ABCC10 and LLC-ABCG2 cells exhibited significantly lower intracellular accumulation of gefitinib at concentration of 1 and 10 μmol/L than the LLC-WT cells. However, at the higher concentrations of 20 and 50 μmol/L, reduced cellular gefitinib accumulation was only observed in LLC-ABCC10 cells, not in LLC-ABCG2 cells, likely due to inhibition of ABCG2 activity at higher concentrations (Ozvegy-Laczka et al., 2004; Leggas et al., 2006; Figure 9).

### Influence of ABCC10 on gefitinib sensitivity in NSCLC xenograft mice models

**Figure 10:**
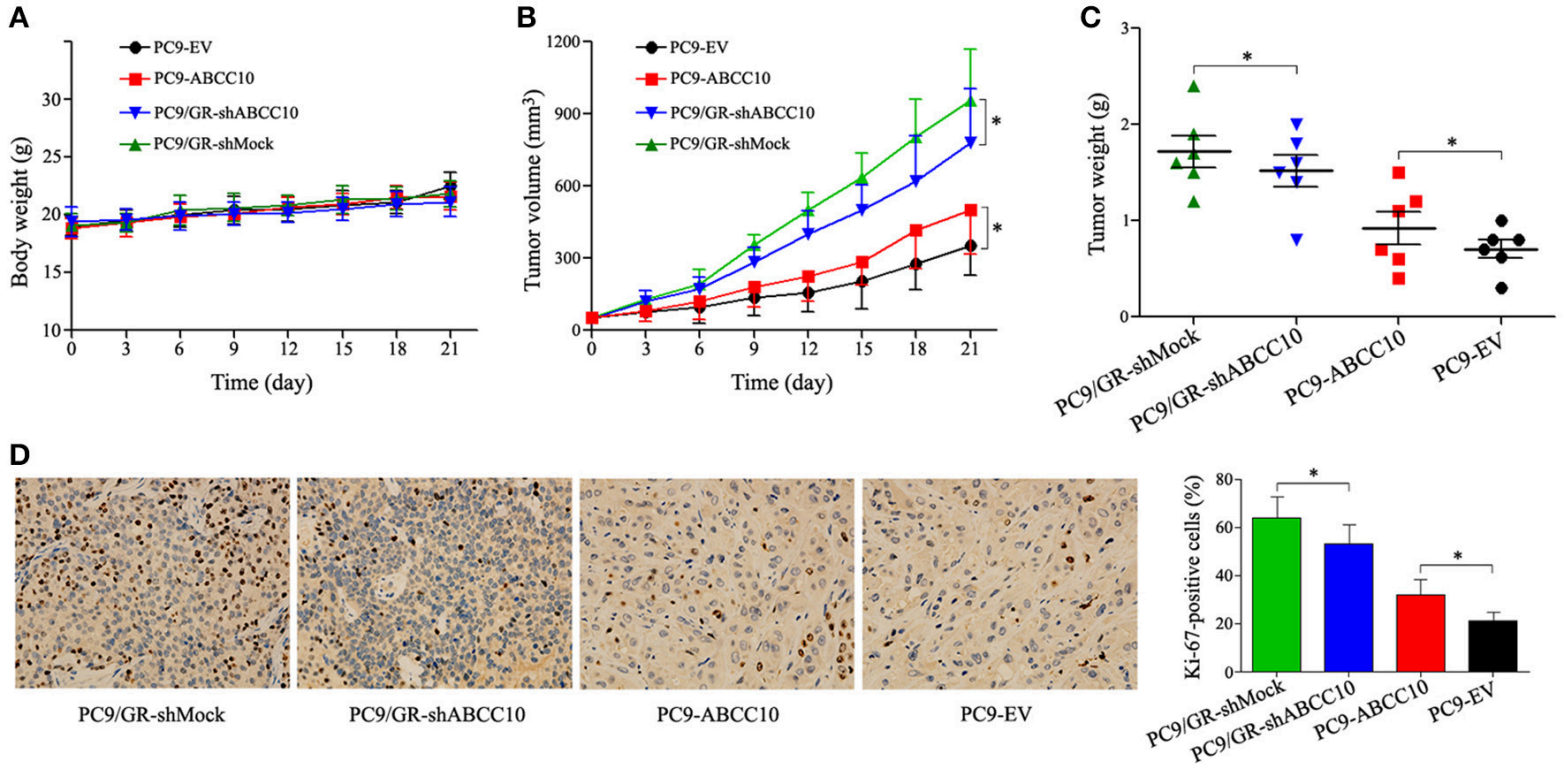
Effect of ABCC10 on gefitinib sensitivity in PC9 xenograft mice model. Nude mice were implanted subcutaneously with the indicated cell lines and were treated with gefitinib (30 mg/kg/day) for 3 weeks (*n* = 6 per group). **(A)** Average body weight curves of Balb/c-nude mice. **(B)** TV was measured as indicated in Materials and methods. **(C)** Tumor weights were analyzed at the end of experiment. **(D)** Proliferation was detected using anti-Ki-67 antibody. Graphs show the percentage of Ki-67-positive cells in each group. Photos of IHC staining are representative of at least 10 similar observations (× 400). *P < 0.05 compared to the PC9/GR-shMock or PC9-EV group.

To further determine whether ABCC10 could reduce the efficacy of gefitinib *in vivo*, PC9 xenograft models were established in female nude mice. Four cell lines, PC9-ABCC10, PC9-EV, PC9/GR-shABCC10, and PC9/GR-shMock were injected subcutaneously into mice, and the mice began receiving gefitinib treatment when tumor volumes reached 50 mm^3^. After the 3-week treatment period, no significant alterations on average body weight were observed among groups (Figure 10A). Compared with PC9-EV group, the average TV and tumor weight showed 1.4-fold (231.4 ± 94.5 mm^3^ vs. 168.3 ± 77.7 mm^3^) and 1.3-fold (0.9 ± 0.3 g vs. 0.7 ± 0.2 g) increases in PC9-ABCC10 group. Similarly, compared with PC9/GR-shMock group, ABCC10 knockdown significantly decreased the average TV and tumor weight by 19% (365.8 ± 96.5 mm^3^ vs. 452.1 ± 86.3 mm^3^) and 12% (1.5 ± 0.4 g vs. 1.7 ± 0.5 g) in PC9/GR-shABCC10 group, respectively (Figures 10B,C). Additionally, we detected the expression of Ki-67, a well-known proliferation marker in NSCLC samples (Tabata et al., 2014). After ABCC10 overexpression, the percentage of Ki-67-positive cells was significantly increased from 21.1% in PC9-EV group to 30.8% in PC9-ABCC10 group. While after ABCC10 knockdown, the percentage of Ki-67-positive cells was markedly decrease from 64.5% in PC9/GR-shMock group to 53.4% in PC9/GR-shABCC10 group (Figure 10D). These results suggested that ABCC10 could reduce the sensitivity of NSCLC cells to gefitinib *in vivo*, and inhibition of ABCC10 expression could partially restore the sensitivity to gefitinib.

## Discussion

ABC transporters play an important role in the absorption, distribution, and elimination of a wide variety of drugs in clinical use, including anticancer chemotherapeutic agents. Numerous studies has confirmed that ABC transporters play an essential role in the development of MDR to chemotherapy. These results lead to the development of three generations of ABC inhibitors. However, despite a few early successes in preclinical studies, these inhibitors failed to improve the effectiveness of chemotherapy in clinical trials due to high toxicity and poor potency (Jaramillo et al., 2018; Mohammad et al., 2018; Robey et al., 2018). The toxicity of these inhibitors is mainly attributed to the inhibition of transporter expressed in normal tissues, and the cross reactivity with other ABC transporters or metabolism enzymes. For example, ABCB1 inhibitor valspodar was found to increase anticancer drug exposure through cytochrome P450 3A4 (CYP3A4) inhibition, while elacrida and tariquidar were found to inhibit both ABCB1 and ABCG2 (de Bruin et al., 1999; Bates et al., 2001; Kannan et al., 2011). Recent developments in tumor-targeted drug delivery systems can be a promising approach to overcoming the side effects of these inhibitors (Binkhathlan and Lavasanifar, 2013; Callaghan et al., 2014). As for the insufficient potency of these inhibitors, one of the main causes may be the lack of full understanding of drug-transporter interactions in MDR. As we all know, one drug may be transported by multiple transporters. In MDR, more than one ABC transporter is likely to be involved in reducing drug accumulation, and the relative importance of these transporters is likely to vary among tumors (Tamaki et al., 2011). Since the expression of ABC transporters continue to be linked to poor outcome in clinic, more efforts should be devoted to investigate the functional aspects of ABC transporters in MDR. With these knowledge, even if the response to chemotherapy cannot be improved by ABC transporter inhibitor, we also can predict clinical response to certain drugs more accurately (Robey et al., 2018).

Recent research has suggested that some ABC transporters may alter the bioavailability of gefitinib at both the cellular and systemic levels, affecting the drug-target interaction and drug sensitivity (Beretta et al., 2017). But very little information is available till now. In the present study, we first established two NSCLC cell lines with acquired resistance to gefitinib, and aimed to identify more ABC transporters potentially involved in the mechanism of gefitinib resistance. In our developed gefitinib-resistant cells, either *EGFR T790M* mutation or amplification of the *MET* proto-oncogene was observed (data not shown). To the best of our knowledge, this is the first work that shows the differential expression of 48 protein coding ABC transporters between gefitinib-sensitive and -resistant NSCLC cells. The RNA-Seq data analysis revealed that most of these transporters did not exhibit significant changes in their expression levels, only the expression levels of *ABCG2* and *ABCC10* transcripts were significantly changed in both EGFR mutant (PC9) and wild-type (H292) NSCLC cell lines simultaneously. The significantly elevated ABCG2 expression in acquired gefitinib-resistant NSCLC cells has been shown previously both *in vitro* and *in vivo* (Usuda et al., 2007; Zhu et al., 2015), but the involvement of ABCC10 in the acquired resistance to EGFR-TKIs has not been reported so far. In this study, we found that both total and cellular membrane ABCC10 protein levels were significantly increased in acquired gefitinib-resistant NSCLC cells, and the increase of membrane ABCC10 level was more obvious than that of the total ABCC10 level. These results imply that some factors may influence the processes of biogenesis and traffic of ABCC10 in NSCLC cells with acquired resistance to gefitinib. To date, mechanisms that regulate ABCC10 expression are largely unknown. Two transcription factors, specificity protein 1 (Sp1) and E2 factor (E2F), as well as NIMA-related expressed kinase 2 (NEK2) have been shown to be involved in the ABCC10 expression regulation (Dabrowska and Sirotnak, 2004; Wu et al., 2017). In addation, some microRNAs, such as several members of let-7 family are referred to downregulate the ABCC10 level (Borel et al., 2012; Wu et al., 2016). Interestingly, previous studies have suggested that many microRNAs play important role in gefitinib resistance, and may also take part in the process of protein membrane trafficking (Sun et al., 2015; Zang et al., 2017). Therefore, regulatory factors, especially microRNAs, that are likely to influence the biogenesis and traffic of ABCC10 in NSCLC cells with acquired resistance to gefitinib warrant further investigation.

Next, we aimed to explore the role of ABCC10 in acquired resistance to gefitinib in NSCLC. We found that ABCC10 decreased the intracellular gefitinib concentration, prevented NSCLC cells from gefitinib-induced growth inhibition and apoptosis *in vitro*. And as expected, ABCC10 also enhanced tumor growth in gefitinib-treated NSCLC xenograft models. Therefore, we hypothesized that ABCC10 might decrease gefitinib sensitivity through pumping out gefitinib from NSCLC cells. To further address this issue, we performed *in vitro* transport study of gefitinib at submicromolar concentration in ABCC10-overexpressing LLC-PK1 cell monolayers. We found that ABCC10 could actively pump gefitinib out of cells, with an ER value of 7.8. According to a white paper published by International Transporter Consortium (ITC), a compound is considered a potential substrate of apically localized transporters if its transport ratio is ≥2 (International Transporter Consortium et al., 2010). Moreover, cepharanthine, an ABCC10 inhibitor, almost completely inhibited gefitinib efflux from LLC-ABCC10 cells. Based on these results, we conclude that, for the first time, ABCC10 actively mediates the transport of gefitinib, and is involved in acquired resistance to gefitinib in NSCLC cells.

To our knowledge only three members of ABC transporter subfamily (i.e., ABCG2, ABCB1, and ABCC1) have been examined with respect to their gefitinib efflux activities so far. Unfortunately, previous study has shown that gefitinib exhibits little or no affinity for ABCB1 and ABCC1 (Ozvegy-Laczka et al., 2004). Considering no changes in the levels of these two transporters were observed in our gefitinib-resistant NSCLC cells, we come to a conclusion that ABCB1 and ABCC1 are unlikely to play a role in the development of acquired resistance to gefitinib in NSCLC. Although it is well established that gefitinib is able to interact with ABCG2, there is, however, controversy over whether gefitinib is a substrate or inhibitor of ABCG2. Some studies have shown that gefitinib is actively extruded by ABCG2, while others indicate that gefitinib is an inhibitor but not a substrate of ABCG2. It is now generally believed that gefitinib can be efflux by ABCG2 at submicromolar concentrations, whereas at higher concentrations it is likely to act as an inhibitor (Ozvegy-Laczka et al., 2004; Leggas et al., 2006; Azzariti et al., 2010; Hegedüs et al., 2012; Beretta et al., 2017). In the present study, we also observed the ABCG2-mediated transport of gefitinib at submicromolar concentration, the unbound plasma level of gefitinib. We found an active efflux of gefitinib by ABCG2, with an ER value of 10.3. This indicates that at submicromolar concentration, the ABCG2-mediated gefitinib transport activity is higher than that of ABCC10. Consistently, we found a lower intracellular gefitinib accumulation in the LLC-ABCG2 cells than in the LLC-ABCC10 cells after 1 μmol/L gefitinib incubation. However, the intracellular gefitinib accumulation in the LLC-ABCG2 cells tend to be higher than in the LLC-ABCC10 cells after 10 μmol/L gefitinib incubation. And at more higher gefitinib concentrations, no significant decrease in intracellular gefitinib concentration could be observed in the LLC-ABCG2 cells compared with LLC-WT cells, but still could be detected in the LLC-ABCC10 cells. These results indicate that ABCG2-mediated gefitinib efflux activity will be decreased with the increase of gefitinib concentration, but ABCC10 can activity extrude gefitinib even at concentration as high as 50 μmol/L. Considering the gefitinib concentration in NSCLC tumor tissues is more than 40-fold higher than plasma levels, we speculate that ABCC10 may play a more important role than ABCG2 in acquired resistance to gefitinib *in vivo* (McKillop et al., 2005; Haura et al., 2010).

In summary, our results show that ABCC10 is overexpressed in NSCLC cells with acquired resistance to gefitinib, independent of EGFR mutation status. As a substrate, gefitinib can be actively transported by ABCC10, even at concentration as high as 50 μmol/L. ABCC10-mediated gefitinib efflux leads to a decrease in the intracellular accumulation, as well as antitumor efficacy of gefitinib in NSCLC *in vitro* and *in vivo*. This study shed more light on the factors that influence acquired resistance to gefitinib, and proposes that ABCC10 can be as a novel predictive marker, as well as a potential therapeutic target for gefitinib acquired resistance in NSCLC.

## Author contributions

HZha, YH, and JS performed the experiments and analyzed the data. HZha and YD interpreted the data and drafted the manuscript. LW and HZho designed the study, supervised the work and wrote the final version of the manuscript. All authors agree to be accountable for the content of the work.

### Conflict of interest statement

The authors declare that the research was conducted in the absence of any commercial or financial relationships that could be construed as a potential conflict of interest.
