# Supplementary material for: ABCC10 Plays a Significant Role in the Transport of Gefitinib and Contributes to Acquired Resistance to Gefitinib in NSCLC
Source: Front Pharmacol. 2018 Nov 20;9:1312. doi: 10.3389/fphar.2018.01312 (PMC6256088; doi:10.3389/fphar.2018.01312)
Supplement: Supplementary file 1 [file Table_1.DOC]

**Supplementary Table 1.** The expression of ABC transporter-encoding genes in PC9/GR cells relative to parental PC9 cells

| **Gene** | **Accession No.** | **PC-9** | **PC-9/GR** | **Fold Change** | **P value** |
| --- | --- | --- | --- | --- | --- |
| ABCA1 | NM_005502.3 | 783.57 | 4214.33 | 5.38 | 0.02^*^ |
| ABCA2 | NM_001606.4 | 281.92 | 408.12 | 1.45 | 0.60 |
| ABCA3 | NM_001089.2 | 645.72 | 715.90 | 1.11 | 0.86 |
| ABCA4 | NM_000350.2 | 188.64 | 597.22 | 3.17 | 0.09 |
| ABCA5 | NM_018672.4 | 144.07 | 449.61 | 3.12 | 0.13 |
| ABCA6 | XM_006721791.2 | 0 | 4.82 | Inf | 0.49 |
| ABCA7 | XM_011527628.1 | 52.86 | 92.62 | 1.75 | 0.63 |
| ABCA8 | NM_001288985.1 | 0 | 0 | - | - |
| ABCA9 | XM_005256937.3 | 3.11 | 0.96 | 0.31 | 0.83 |
| ABCA10 | NM_080282.3 | 52.86 | 133.15 | 2.52 | 0.38 |
| ABCA12 | XM_011510951.1 | 287.10 | 263.40 | 0.92 | 0.91 |
| ABCA13 | XM_011515130.1 | 0 | 24.12 | Inf | 0.17 |
| ABCB1 | NM_000927.4 | 2.07 | 1.93 | 0.93 | 1 |
| ABCB2 | NM_000593.5 | 1622.07 | 1702.91 | 1.05 | 0.92 |
| ABCB3 | NM_000544.3 | 310.94 | 348.305 | 1.12 | 0.87 |
| ABCB4 | NM_018849.2 | 0 | 0 | - | - |
| ABCB5 | NM_178559.5 | 1.04 | 0.96 | 0.93 | 1 |
| ABCB6 | NM_005689.2 | 509.94 | 519.07 | 1.018 | 0.98 |
| ABCB7 | NM_001271696.1 | 886.18 | 822.03 | 0.93 | 0.89 |
| ABCB8 | NM_007188.4 | 629.13 | 301.99 | 0.48 | 0.26 |
| ABCB9 | XM_011538098.1 | 63.22 | 98.41 | 1.56 | 0.69 |
| ABCB10 | NM_012089.2 | 1197.12 | 1137.52 | 0.95 | 0.92 |
| ABCB11 | NM_003742.2 | 0 | 0 | - | - |
| ABCC1 | NM_004996.3 | 1309.05 | 1656.59 | 1.27 | 0.63 |
| ABCC2 | NM_000392.4 | 3349.85 | 6535.68 | 1.95 | 0.10 |
| ABCC3 | NM_003786.3 | 4281.63 | 7453.23 | 1.74 | 0.16 |
| ABCC4 | NM_005845.4 | 1468.67 | 693.70 | 0.47 | 0.16 |
| ABCC5 | XM_005247059.3 | 1112.13 | 1941.22 | 1.75 | 0.26 |
| ABCC6 | XM_011522479.1 | 18.66 | 37.63 | 2.02 | 0.68 |
| ABCC7 | NM_000492.3 | 0 | 0 | - | - |
| ABCC8 | XM_011520333.1 | 0 | 0.96 | Inf | 1 |
| ABCC9 | NM_020297.3 | 348.25 | 200.68 | 0.58 | 0.46 |
| ABCC10 | NM_033450.2 | 157.62 | 918.93 | 5.83 | 0.02^*^ |
| ABCC11 | NM_032583.3 | 10.36 | 3.869 | 0.37 | 0.74 |
| ABCC12 | NM_033226.2 | 0 | 0 | - | - |
| ABCD1 | NM_000033.3 | 569.02 | 714.93 | 1.26 | 0.70 |
| ABCD2 | NM_005164.3 | 0 | 0.96 | Inf | 1 |
| ABCD3 | NM_002858.3 | 3084.52 | 2775.78 | 0.90 | 0.8 |
| ABCD4 | XM_005267941.3 | 173.09 | 79.12 | 0.46 | 0.41 |
| ABCE1 | NM_002940.2 | 2177.61 | 5762.86 | 2.65 | 0.04^*^ |
| ABCF1 | NM_001025091.1 | 1669.74 | 2450.64 | 1.47 | 0.41 |
| ABCF2 | NM_005692.4 | 1087.25 | 1933.50 | 1.78 | 0.24 |
| ABCF3 | NM_018358.2 | 1050.97 | 1694.22 | 1.61 | 0.34 |
| ABCG1 | NM_004915.3 | 138.35 | 274.01 | 1.98 | 0.21 |
| ABCG2 | NM_004827.2 | 564.41 | 2280.83 | 4.04 | 0.03^*^ |
| ABCG4 | NM_001142505.1 | 5.18 | 0 | 0 | 0.46 |
| ABCG5 | NM_022436.2 | 0 | 0 | - | - |
| ABCG8 | NM_022437.2 | 0 | 0 | - | - |

Data are represented as mean values calculated from three-repeated experiments. ^*^*P* < 0.05.
