# Supplementary material for: ABCC10 Plays a Significant Role in the Transport of Gefitinib and Contributes to Acquired Resistance to Gefitinib in NSCLC
Source: Front Pharmacol. 2018 Nov 20;9:1312. doi: 10.3389/fphar.2018.01312 (PMC6256088; doi:10.3389/fphar.2018.01312)
Supplement: Supplementary file 2 [file Table_2.doc]

**Supplementary Table 2.** The expression of ABC transporter-encoding genes in H292/GR cells relative to parental H292 cells

| **Gene** | **Accession No.** | **H292** | **H292/GR** | **Fold Change** | **P value** |
| --- | --- | --- | --- | --- | --- |
| ABCA1 | NM_005502.3 | 667.68 | 831.86 | 1.25 | 0.56 |
| ABCA2 | NM_001606.4 | 299.55 | 445.18 | 1.49 | 0.41 |
| ABCA3 | NM_001089.2 | 264.25 | 304.39 | 1.15 | 0.79 |
| ABCA4 | NM_000350.2 | 6.05 | 3.97 | 0.66 | 0.93 |
| ABCA5 | NM_018672.4 | 547.66 | 495.74 | 0.91 | 0.82 |
| ABCA6 | XM_006721791.2 | 0 | 0.99 | Inf | 1 |
| ABCA7 | XM_011527628.1 | 33.28 | 44.62 | 1.34 | 0.82 |
| ABCA8 | NM_001288985.1 | 0 | 0 | - | - |
| ABCA9 | XM_005256937.3 | 0 | 0 | - | - |
| ABCA10 | NM_080282.3 | 171.46 | 101.13 | 0.59 | 0.46 |
| ABCA12 | XM_011510951.1 | 569.85 | 615.71 | 1.08 | 0.85 |
| ABCA13 | XM_011515130.1 | 8.07 | 0 | 0 | 0.36 |
| ABCB1 | NM_000927.4 | 4.03 | 1.98 | 0.49 | 0.89 |
| ABCB2 | NM_000593.5 | 2721.16 | 3258.03 | 1.20 | 0.46 |
| ABCB3 | NM_018833.2 | 2567.85 | 1998.84 | 0.78 | 0.34 |
| ABCB4 | NM_018849.2 | 0 | 0 | - | - |
| ABCB5 | NM_178559.5 | 7.06 | 0.99 | 0.14 | 0.57 |
| ABCB6 | NM_005689.2 | 151.29 | 118.98 | 0.79 | 0.74 |
| ABCB7 | NM_001271696.1 | 410.49 | 596.88 | 1.45 | 0.39 |
| ABCB8 | NM_007188.4 | 723.15 | 881.43 | 1.22 | 0.59 |
| ABCB9 | XM_011538098.1 | 30.26 | 40.65 | 1.34 | 0.83 |
| ABCB10 | NM_012089.2 | 1990.94 | 2119.80 | 1.06 | 0.82 |
| ABCB11 | NM_003742.2 | 0 | 0.99 | Inf | 1 |
| ABCC1 | NM_004996.3 | 927.90 | 1421.80 | 1.53 | 0.19 |
| ABCC2 | NM_000392.4 | 140.19 | 182.43 | 1.30 | 0.70 |
| ABCC3 | NM_003786.3 | 111.95 | 75.35 | 0.67 | 0.64 |
| ABCC4 | NM_005845.4 | 290.47 | 672.23 | 2.31 | 0.04^*^ |
| ABCC5 | XM_005247059.3 | 91.78 | 63.46 | 0.69 | 0.68 |
| ABCC6 | XM_011522479.1 | 8.07 | 15.86 | 1.97 | 0.76 |
| ABCC7 | NM_000492.3 | 0 | 0 | - | - |
| ABCC8 | XM_011520333.1 | 0 | 0 | - | - |
| ABCC9 | NM_020297.3 | 748.37 | 410.48 | 0.55 | 0.14 |
| ABCC10 | NM_033450.2 | 92.78 | 603.84 | 6.50 | 0.03^*^ |
| ABCC11 | NM_032583.3 | 0 | 0 | - | - |
| ABCC12 | NM_033226.2 | 0 | 0 | - | - |
| ABCD1 | NM_000033.3 | 188.61 | 247.87 | 1.31 | 0.65 |
| ABCD2 | NM_005164.3 | 0 | 0 | - | - |
| ABCD3 | NM_002858.3 | 2409.51 | 34891.31 | 2.03 | 0.04^*^ |
| ABCD4 | XM_005267941.3 | 61.52 | 66.43 | 1.08 | 0.95 |
| ABCE1 | NM_002940.2 | 7779.21 | 8408.82 | 1.08 | 0.70 |
| ABCF1 | NM_001025091.1 | 3104.42 | 2775.18 | 0.89 | 0.65 |
| ABCF2 | NM_005692.4 | 444.79 | 486.82 | 1.09 | 0.84 |
| ABCF3 | NM_018358.2 | 1271.82 | 1270.10 | 1 | 1 |
| ABCG1 | NM_004915.3 | 75.64 | 273.65 | 3.62 | 0.04^*^ |
| ABCG2 | NM_004827.2 | 261.75 | 1130.76 | 4.32 | 0.04^*^ |
| ABCG4 | NM_001142505.1 | 0 | 1.98 | Inf | 0.67 |
| ABCG5 | NM_022436.2 | 0 | 0 | - | - |
| ABCG8 | NM_022437.2 | 0 | 0 | - | - |

Data are represented as mean values calculated from three-repeated experiments. ^*^*P* < 0.05.
